# Age‐specific incidence and prevalence of childhood type 1 diabetes: Development over four decades in Southwest Germany

**DOI:** 10.1111/dme.70215

**Published:** 2026-01-06

**Authors:** Julian Ziegler, Andreas Neu, Stefan Ehehalt, Roland Schweizer, Klaus Dietz

**Affiliations:** ^1^ Department of Pediatric and Adolescent Medicine University Hospital Tuebingen Tuebingen Germany; ^2^ Health Department of the City of Stuttgart Stuttgart Germany; ^3^ Department of Medical Biometry University of Tuebingen Tuebingen Germany

**Keywords:** Covid‐19‐pandemic, epidemiology, incidence rate of T1D, prevalence rate of T1D, Type‐1‐diabetes in children and adolescents

## Abstract

**Aims:**

This study evaluated the incidence and prevalence of type 1 diabetes in children and adolescents in Baden–Wuerttemberg over 38 years considering various subgroups.

**Methods:**

Data were drawn from the German Diabetes Registry (DIARY) for 11,797 children aged <15 years with clinically diagnosed type 1 diabetes. Incidence rates were calculated per 100,000 person‐years and presented as crude, age‐ and sex‐specific rates, including annual percent changes (APC) from 1987 to 2024.

**Results:**

In 1987, the crude incidence was 10.1 per 100,000 children per year, rising to 20.2 per 100,000 children per year in 2024. Between 1987 and 2008, the incidence increased steadily at 4.0% annually, followed by a plateau through 2024 despite demographic shifts including declining birth rates and increased immigration. The peak incidence occurred during the COVID‐19 pandemic, reaching 31.1 per 100,000 children per year in 2021, before returning to 20.2 per 100,000 children per year by 2024. The highest subgroup incidence was found in boys aged 10–14 years at 25.4 per 100,000 children per year. A marked increase during the pandemic was seen mainly in children under 10 years. The estimated prevalence as of 31 December 2024 was 0.154%.

**Conclusions:**

Incidence rates have doubled since the first documentation in 1987. Since 2008, the rates have remained stable at a high level without further increase. The long‐term trend suggests a levelling off in incidence, with pandemic‐related increases limited primarily to younger children.

**What's new**

**What is already known?**

- The incidence rate remained stable in Scandinavian countries for 20 years. Germany has one of the highest rates of type 1 diabetes in Europe. During the pandemic, the incidence rate among children and adolescents increased significantly.

**What this study has found?**

- The incidence rate in Germany increased steadily by 4% until 2007, after which it remained stable until the outbreak of COVID‐19. After the pandemic, the incidence rate fell rapidly, indicating that the initial increase was temporary. The highest incidence rate was found among boys aged 10 to 14.

**What are the implications of the study?**

- We present current epidemiological figures and findings on possible triggers that may influence the autoimmune process in type 1 diabetes.

## 1 INTRODUCTION

Rising incidence rates of type 1 diabetes in children have been reported from different parts of the world over the past decades. Compared to other parts of the world, the incidence rates in Europe are the highest in the world.^1^ Despite strong regional differences, the number of new cases in Europe increased by an average of 3.4% per year, suggesting a doubling of the incidence rate in about 20 years.^2^ However, declining incidence rates have been reported from the Scandinavian countries such as Finland and Norway,^3^, ^4^ and there is evidence of a plateauing of the incidence curve in Western Australia^5^ and also for Scotland,^6^ the country with the second‐highest incidence rate after Finland.

During the COVID‐19 pandemic, changes in incidence were observed in many countries.^7^ Significantly higher incidence rates were also observed in Germany: A 16% (95% CI 10–23) higher incidence rate in 2020 and a 28% (95% CI 18–39) higher incidence rate in 2021 compared to the year before the pandemic.^8^ The impact of the COVID‐19 pandemic on the long‐term trend in the prevalence of type 1 diabetes has been discussed in detail.^9^

There is no national diabetes registry for the whole of Germany. However, there are three operational regional registries for incident cases of type 1 diabetes in childhood,^10^ each of which participates in the EURODIAB network.^11^ The oldest of these registries is the Baden–Wuerttemberg Diabetes Registry (DIARY), which has been continuously recording new cases in the age group <15 years for 38 years since 1987. With 11,797 case registrations from 1987 to 2024, DIARY is one of the oldest and largest registries in the world.

Baden–Wuerttemberg is a federal state in southwestern Germany with an area of 35,751 km^2^, which is 10% of the total area of the country. With a population of 11,245,898 people (31.12.2024), the population of Baden–Wuerttemberg represents 13.46% of the total population of Germany (83,577,140). As of 31 December 2024, 1.62 million of the population were children under the age of 15. This corresponds to 13.96% of all German children in this age group.^12^ Declining birth rates on the one hand and increasing numbers of immigrants on the other have influenced and changed the demographic structure, especially in the last decade.

In the light of this background, the present long‐term observation analyses the incidence trend and the annual change in incidence for the age groups ≤4, 5–9, and 10–14 years, including the period of the corona pandemic. The impact of changing incidence rates and demographic changes on prevalence was also considered. The changes that occurred before, during and after the corona pandemic were discussed in detail elsewhere.^13^ The present focus is on the prevalence and subgroup analyses of incidence.

## 2 RESEARCH DESIGN AND METHODS

The analysis was based on 11,797 cases diagnosed in the German state of Baden–Wuerttemberg over a 38‐year period from 1987 to 2024. Children under 15 years of age with diagnosed type 1 diabetes were usually referred to one of the 32 hospitals forming the DIARY (Diabetes Registry) network. All paediatric departments (*n* = 31) in Baden–Wuerttemberg and one diabetes centre contribute to this registry. People with a monogenetic form of diabetes or aged under 6 months were excluded from the registry.

Population data refer to the 1987 and 2011 censuses and annual updates provided by the Federal Statistical Office in Wiesbaden, Germany. Because medical care in Baden–Wuerttemberg may be sought by people from neighbouring regions and vice versa, we included all registered cases in our analysis, regardless of whether they were residents of Baden–Wuerttemberg or not.

The degree of ascertainment was calculated according to the capture–mark–recapture method. A survey was conducted as a secondary data source at meetings of the Diabetiker Bund Baden–Württemberg (a people organization) in 1995, 2010 and 2013. This resulted in a completeness rate of over 97%. Due to the corona pandemic, it was not possible to repeat the survey from 2020 to 2023. Therefore, a comparison was made with the Diabetes Prospective Follow‐up (DPV) registry in 2025, which revealed a constant high degree of ascertainment of the registry.

Standardization for sex and age was performed according to the EURODIAB criteria. The direct method of standardization was used, using a standard population with equal numbers in each subgroup defined by age and/or sex. We present crude and standardized incidence rates based on the number of cases recorded and stratified by age (≤4, 5–9, 10–14 years) and sex (girls and boys) groups for each of 38 years.

The prevalence of children less than 15 years at the beginning of a year between 2003 and 2025 is the cumulative number of cases recorded up to that date. The prevalence rate before 2003 cannot be determined from our data, as data from people diagnosed with diabetes before 1987 are missing.

A Poisson distribution was assumed for the calculation of 95% confidence intervals. The 95% confidence intervals for the standardized rates were calculated using a method based on the gamma distribution. We describe the mean age at onset of diabetes with respect to cohorts instead of calendar years because of lower variability.

## 3 RESULTS

### 3.1 Demographic data, population at risk

At the end of 2024, there are 1,621,806 children and adolescents under the age of 15 in Baden–Wuerttemberg and 11,619,895 in Germany as a whole. Children in this age group are considered to be children at risk. Initially, there was a continuous increase in the population from 1987 to the late 1990s. Subsequently, the population of children and adolescents under 15 years of age decreased until it reached a minimum in 2014, followed by a continuous increase in the number of children and adolescents in Baden–Wuerttemberg, which continues until today. This trend affects boys and girls equally.

### 3.2 Overall incidence rate

In 1987, the crude incidence was 10.1 [95% CI 8.5–11.9] per 100,000 children per year based on the population figures above, the standardized rate was 10.2 [95% CI 8.6–12.0] per 100,000 children per year. In 2022, the incidence peaked with a crude rate of 31.1 [95% CI 28.4–34.0] per 100,000 children per year and a standardized rate of 31.2 [95% CI 28.5–34.0] per 100,000 children per year. Finally, in 2024, the crude rate was 20.2 [95% CI 18.0–22.5] per 100,000 children per year, and the standardized rate was 20.1 [95% CI 18.0–22.4] per 100,000 children per year, which is double the number compared to 1987.

The rate for boys in 2024 was 21.5 [95% CI 18.5–24.8] per 100,000 children per year, slightly higher than the rate for girls, which was 18.8 [95% CI 15.9–22.0] per 100,000 children per year.

### 3.3 Incidence trend

**FIGURE 1:**
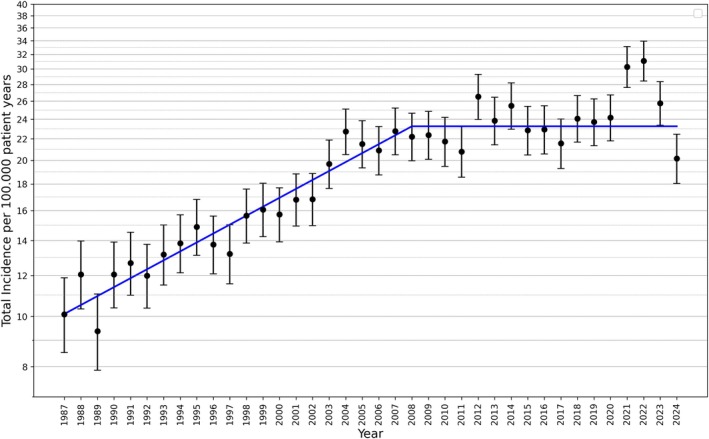
Incidence trend over 38 years 1987–2024 (crude incidence rates with 95% CI).

The incidence rate increased by 4.0% [95% CI 3.6–4.6%] per year until 2008. Subsequently, the incidence stayed constant. The pandemic years 2021 and 2022 were excluded for parameter estimation because they are not representative of the entire observation period. During these two years, the incidence increased significantly by 32% (*p* < 0.0001) for the total population, but not for all subgroups. Children and adolescents older than 10 years did not show a significant increase in type 1 diabetes mellitus incidence during the pandemic. The incidence trend over time is shown in Figure 1 on a logarithmic scale.

### 3.4 Sex and age distribution

Boys represented 53.4% (*n* = 6298) and girls 46.6% (*n* = 5499) of the registered cases. The age group ≤ 4 years comprised 23.35%, 5–9 years comprised 35.81% and 10–14 years comprised 40.84% of the children at diagnosis, with no significant difference in sex distribution among the age groups.

**FIGURE 2:**
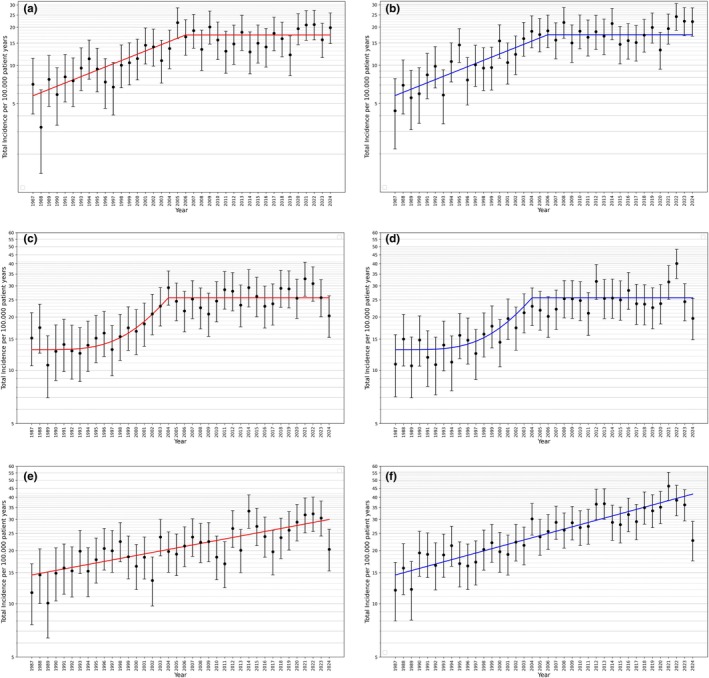
Incidence trends for different age groups (crude incidence rates with 95% CI); (a) and (b) age group ≤4 years, (c) and (d) age group 5–9 years, (e) and (f) age group 10–14 years; figures on the left ((a), (c) and (e)) refer to girls, figures on the right ((b), (d) and (f)) refer to boys.

The highest incidence rates were found in the age group 10–14 years for boys, and the lowest in the age group ≤ 4 years for both boys and girls. The figures are shown in Table 1. The annual trend in incidence for boys and girls in the different age groups is shown in Figure 2.

**TABLE 1:** The total number of cases from 1987 to 2024 and the crude incidence rates for boys and girls in different age groups are given. The 95% confidence interval is given as the upper and lower limits (Inc UL and LL).

Age (years)	Sex	Cases	Inc	Inc LL	Inc UL
≤4	Girls	1291	13.6	12.8	14.3
5–9	Girls	2100	21.7	20.8	22.7
10–14	Girls	2108	21.4	20.5	22.3
≤4	Boys	1463	14.6	13.9	15.4
5–9	Boys	2125	20.9	20.0	21.8
10–14	Boys	2710	26.1	25.1	27.1

The course of the incidence rate in the subgroups of children and adolescents is different.

While the incidence rate in the age groups ≤4 years and 5–9 years remained constant from 2006 and 2004, respectively, with a rate of 17.4 and 25.7 per 100,000 children, respectively, the incidence rate in adolescents aged 10–14 years continues to rise exponentially. In the period from 1987 to 2004, the incidence increase was also exponential in the youngest age group with an annual percent change (APC) of 6.0% (95% CI 5.2–6.8%), while in the middle age group, a strong increase was only seen in the years 1994 to 2004, which can best be described with a power function.

The baseline incidence in the age group 10–14 years was 14.6 (95% CI 13.6–15.5) per 100,000. The APC for girls was 2.0% (95% CI 1.6–2.3%) and 2.9% (95% CI 2.6–3.2%) for boys.

The above‐mentioned increase of 32% in the pandemic years 2021 and 2022 is not equally represented in all age groups. The increase is mainly observed in the age group 5–9 years and was significant in boys ≤4 years and 5–9 years in 2022; (*p* < 0.05).

Onset mean age decreased over time, from 9.3 years in the 1987 cohort to 8.7 years in the 2010 cohort for boys, and from 9.0 years in the 1987 cohort to 8.2 years in the 2010 cohort for girls. This equates to approximately 0.28 months per decade for boys and 0.37 months for girls.

### 3.5 Prevalence

**FIGURE 3:**
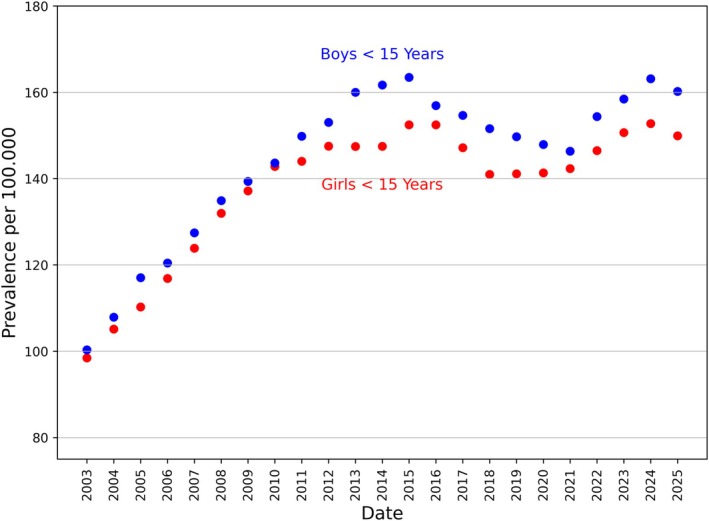
Prevalent cases of type 1 diabetes in children under the age of 15; the prevalence rate before 2003 cannot be determined from our data, as data from people diagnosed with diabetes before 1987 are missing.

The prevalence at the end of our observation period on 31 December 2024 for the group of children under 15 was 2.550. In Baden–Wuerttemberg, 1351 boys and 1199 girls had type 1 diabetes mellitus. This corresponds to a proportion of 0.154% [0.149–0.161%] of the total population of the same age group, that is one in 647 children of the same age group was affected at the end of 2024. Extrapolating to the national population of children under 15 years of age yields 18,454 affected children in Germany. The prevalence over time is shown in Figure 3.

## 4 DISCUSSION

Data on the incidence and prevalence of type 1 diabetes in childhood are available from many parts of the world. Only a few of them—mainly from Europe—contain data for more than 30 years with consistently high completeness.

The incidence figures reported so far from Germany indicate that Germany, represented by three EURODIAB registries, has an incidence rate in an intermediate dimension in Europe. The rate of increase in incidence for Baden–Wuerttemberg was previously reported to be 4.1% for the 2009–2013 period.^2^

Compared with the overall annual increase in Europe over the same period, which was estimated to be 3.4% [95% CI 2.8–3.9%], the increase in incidence was considered to be moderately high. Since 2008, the situation has changed: the incidence has remained more or less constant, mainly due to a flattening of the curve in persons under 10 years of age. This is consistent with observations in Finland,^3^ Norway,^4^ Australia^5^ and Scotland,^6^ where declining incidence has led to a plateau. Adolescents still have the highest incidence rate. This is undoubtedly due to pubertal development, which involves an increase in hormones with a counter‐insulin effect, such as oestrogen, testosterone and growth hormone. Other contributing factors include rapid growth in height and physiological insulin resistance. The decline in the age of diabetes onset is consistent with the earlier puberty onset described by Eckert‐Lind et al. as a secular trend over the last four decades^14^ and corresponds almost exactly to the reported earlier thelarche of approximately three months.

Whether this stabilization of new cases at a high level will be permanent or not remains speculative and points to the need for further registration. One possible explanation could be that the previously reported shift towards younger ages, with the associated increase in incidence, is now coming to an end.

Since the turn of the millennium, the total number of children in Baden–Wuerttemberg and Germany has been declining, reaching a minimum in 2013. It is important to be familiar with this demographic trend to understand why the prevalence calculated in these years was almost as high as in previous years, despite the rising incidence rate. The situation changed in 2014, partly due to the increasing number of immigrants coming to Germany.

During the Corona pandemic, a significantly higher incidence rate was observed in 2021 and 2022. This phenomenon has also been observed by others and attributed to direct effects of the SARS‐CoV‐2 virus or to indirect effects during the pandemic situation, such as weight gain associated with an increased risk of islet autoimmunity.^15^, ^16^ Our long‐term observation shows that the increase during these years was temporary, similar to peaks in previous years (1988, 2004 and 2012). The subsequent incidence rates in 2023 and 2024 are in the range of the pre‐pandemic years. Therefore, the sharp rise in the incidence rate during the Coronavirus pandemic can be viewed as a unique situation brought about by the emergence of the SARS‐CoV‐2 virus and its associated factors. It is possible that people in Stage 2 of the disease progressed to Stage 3 earlier due to the additional stress caused by the SARS‐CoV‐2 virus itself or by consecutive measures such as closure of schools, lockdown and psychological stress. Direct cytotoxic effects of SARS‐CoV‐2 on beta cells could not be observed. Kamrath et al.^17^ ruled out an increased rate of antibody‐negative diabetes, which would be expected in this case. The incidence peaks and troughs of type 1 diabetes point to environmental factors driving diabetic autoimmunity.

Since register analysis is descriptive, we can only identify associations, not draw conclusions. Therefore, the reason why children younger than 10 years had the highest incidence rate during the pandemic remains speculative. However, a faster transition from Stage 2 to Stage 3 diabetes is the most appropriate explanation for this phenomenon. The peak of onset so far has been seen between 10 and 14 years of age, but due to the stress of the corona pandemic, this peak may be shifted towards younger age groups.

However, an increase in the incidence rate among young children was not observed in all European countries. A recent report on incidence rates in Scotland showed no increase in this age group.^18^

The findings of our study may be limited by the fact that people living on the border of Baden–Wuerttemberg who sought treatment at a hospital in a neighbouring federal state were not included. However, due to the high coverage of >97%, this effect can be considered negligible. Uncertainties in diagnosis may also limit the findings, as diagnoses are made in the participating hospitals and cannot be verified by the registry. A sub‐analysis by ethnicity is not possible on an annual basis due to the small number of cases.

The strength of our study is the observation period over 38 years with 11,979 cases included and the high completeness (>97%). The long duration of registration includes the years of the corona pandemic (2021 and 2022), as well as the time after (2023 and 2024). A limitation is certainly the fact that Baden–Wuerttemberg only partially represents Germany. On the other hand, a previous publication^10^ showed that the incidence rates reported from other parts of Germany—the states of North Rhine‐Westphalia and Saxony—were close to those reported from Baden–Wuerttemberg. In view of this observation and the large number of cases included in our study, we consider the incidence and prevalence figures presented to be representative for Germany as a whole, apart from some regional differences.

## AUTHOR CONTRIBUTIONS

All authors were involved in the design and creation of the manuscript. In addition, K.D. was responsible for the statistical analysis. A.N. and J.Z. for data collection and maintaining the registry. All three developed the concept of the work and wrote the first version of the manuscript. R.S. and S.E. provided helpful and important intellectual content for the design and writing of the manuscript and were also responsible for revising the final version of the manuscript for publication. J.Z. is the guarantor of this work and, as such, had full access to all the data in the study and takes responsibility for the integrity of the data and the accuracy of the data analysis.

## FUNDING INFORMATION

No funding has to be reported.

## CONFLICT OF INTEREST STATEMENT

No potential conflicts of interest relevant to this article have to be reported. No artificial intelligence was used.

## Data Availability

The data that support the findings of this study are not openly available due to reasons of sensitivity and are available from the corresponding author upon reasonable request.
